# Clinical outcomes of BRAF plus MEK inhibition in melanoma: A meta‐analysis and systematic review

**DOI:** 10.1002/cam4.2248

**Published:** 2019-08-08

**Authors:** Qingliang Yu, Jiayi Xie, Jiangmiao Li, Yunxin Lu, Liang Liao

**Affiliations:** ^1^ Guangxi Medical University The Guangxi Zhuang Autonomous Region Nanning China

**Keywords:** BRAF inhibition, combination therapy, MEK inhibition, melanoma, monotherapy

## Abstract

**Background:**

Melanoma is a potentially fatal malignancy with poor prognosis. Several recent studies have demonstrated that combination therapy of BRAF and MEK inhibition achieved better curative effect and appeared less toxic effects. We conducted a meta‐analysis to evaluate the efficacy and safety between BRAF inhibition plus MEK inhibition combination therapy and BRAF inhibition monotherapy in melanoma patients.

**Methods:**

We performed the search in PubMed, EMBASE, and the Cochrane Library from January 2010 to January 2019. Inclusion and exclusion of studies, assessment of quality, outcome measures, data extraction, and synthesis were independently accomplished by two reviewers. Revman 5.3 software was used for the meta‐analysis.

**Results:**

Totally, seven randomized controlled trials involving 3146 patients met our inclusion criteria. Comparing the results of combination therapy and monotherapy, combination therapy significantly improved OS (RR = 1.13; 95% CI, 1.08, 1.19; *P* < 0.00001), ORR (RR = 1.36; 95% CI, 1.28, 1.45; *P* < 0.00001), PFS (RR = 0.57; 95% CI, 0.52, 0.63; *P* < 0.00001) and reduced deaths (RR = 0.78; 95% CI, 0.69, 0.88; *P* < 0.0001). Skin‐related adverse events such as hyperkeratosis, cutaneous squamous‐cell carcinoma were less compared with monotherapy. However, gastrointestinal events like nausea, diarrhea, and vomiting were at a higher frequency.

**Conclusion:**

Doublet BRAF and MEK inhibition achieved better survival outcomes over single‐agent BRAF inhibition and occurred less skin‐related events, but gastrointestinal events were more in combination therapy.

## Graphical abstract

We conducted a meta‐analysis to compare chemotherapy in melanoma with combination of BRAF inhibition and MEK inhibition or BRAF inhibition monotherapy. A total of 3146 patients with histologically confirmed metastatic melanoma were included in our meta‐analysis and we found a better overall survival and treatment response. Subgroup analysis demonstrated that combination therapy was related to a more frequent incidence of pyrexia and gastrointestinal events and BRAF inhibition alone was at a high risk of dermatologic diseases.

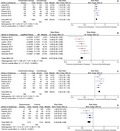

## 1 INTRODUCTION

Melanoma is a severe cutaneous carcinoma with an increasing incidence rate.^1^, ^2^, ^3^ Melanoma accounts for only 2.3% of all cutaneous malignancies, but it leads to the majority of skin‐cancer deaths.^4^, ^5^ The 5‐year survival rate of patients with metastatic melanoma is between 5% and 19%.^6^, ^7^ Melanoma has a strong invasive capability and systematic appearance of acquired resistance, which complicates clinical treatment.^5^, ^7^

Several recent studies 8, 9, 10 have demonstrated that BRAF inhibition combined with MEK inhibition achieved better curative effect vs BRAF monotherapy. BRAF inhibitions (eg, dabrafenib, vemurafenib, encorafenib) plus MEK inhibitions (eg, cobimetinib, trametinib, binimetinib) significantly improved the overall survival (OS), progression‐free survival (PFS) and overall response rate (ORR) in metastatic melanoma patients, as compared with single agent BRAF inhibition.^11^, ^12^ What's more, patients treated with BRAF inhibition alone often developed acquired resistance resulting in discontinuation of monotherapy.^13^, ^14^ And more patients occurred cutaneous squamous‐cell carcinoma or cutaneous hyperkeratosis.^15^ Compared with BRAF inhibition alone, combination therapy delayed the occurrence of acquired resistance and appeared less toxic effects.^16^, ^17^ However, combination therapy was related to a higher incidence of pyrexia and gastrointestinal events (eg, diarrhea, nausea, or vomiting).^13^

Based on existing literature, we try to evaluate the effects and clinical relevant adverse events between combination therapy (BRAF and MEK inhibition) and monotherapy (BRAF inhibition alone).

## 2 MATERIALS AND METHODS

### 2.1 Literature search

The PubMed, EMBASE and the Cochrane Library were searched from January 2010 to January 2019. Search formulas were the following: “BRAF inhibition,” “MEK inhibition,” “melanoma,” “dabrafenib,” “trametinib,” “vemurafenib,” “cobimetinib,” “binimetinib,” “encorafenib.” Article type was not limited for potential studies.

### 2.2 Study selection

The selection criterions were: (1) The study design of literature was randomized controlled trial. (2) Patients in the study were diagnosed with metastatic melanoma. (3) Treatment was BRAF inhibition in combination with MEK inhibition compared with single drug BRAF inhibition. (4) The study results included adverse events (AEs) and efficacy, including overall survival (OS), mortality, progression‐free survival (PFS), and overall response rate (ORR). Exclusion criterions were: (1) The research content was not related to drug efficiency and safety. (2) Melanoma patients without mutations of BRAF V600E or BRAF V600K. (3) The research design was not randomized controlled trial. (4) Result was not available or incomplete.

### 2.3 Extraction of data and quality assessment

Extraction of data was completed by two reviewers independently, according to standardized data‐collection form. Data extraction form consisted of the following: number of patients, median age, male ratio, OS, mortality, ORR, PFS, and adverse events. The adverse events selected subsequently were mainly classified into skin‐related events, gastrointestinal events, and then further analyzed. Most common adverse events were extracted including gastrointestinal events and cutaneous events. Results of both participants were compared, any differences found were discussed and then referred to the original article for correction. Quality assessment was conducted by the Cochrane Collaboration's risk‐of‐bias tool.

### 2.4 Analysis of data

Data analysis was carried out via Revman 5.3. Tests for homogeneity were assessed primarily by *I* ^2^ statistic to determine whether the statistics can be combined. If *I* ^2^ value was less than 25%, it could be considered as a low level heterogeneity. The value between 25% and 50% was significant and indicated that the studies may be homogeneous while *I* ^2^ value more than 50% was insignificant. The risk ratio (RR) was used to estimate the efficacy and safety. 95% confidence interval (95% CI) was calculated to estimate population parameters. Indispensably, *P* value was calculated as a measurement of statistical significance. The results of risk ratio value or 95% CI were not statistically significant unless the *P* value was less than 0.05.

## 3 RESULTS

### 3.1 Literature search and study characteristics

**Figure 1:**
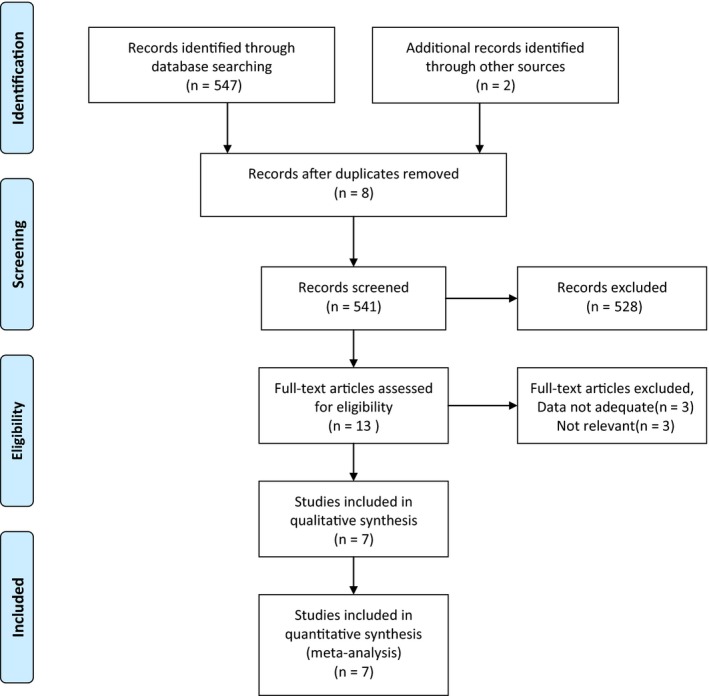
Flow diagram of studies selection

Seven randomized controlled trials were selected in total.^8^, ^9^, ^11^, ^12^, ^13^, ^18^, ^19^ Our initial literature search found a total of 549 relevant citations. After duplication, 541 studies were included. Subsequently, 528 of 541 studies were excluded because their titles and abstracts did not fulfill our inclusion criteria. Six of the remaining 13 studies were further discarded after full‐text assessing. Details about selection of studies were illustrated in Figure 1. Of all eligible studies, five were randomized phase 3 trials and one was randomized phase 1, 2 trial and one was unknown. All these included studies were carried out between 2012 and 2018. A total of 3146 patients with histologically confirmed metastatic melanoma were included in assessment and 2046 patients were at stage M1c. All studies were consistent with the principle of combination therapy (MEK inhibition plus BRAF inhibition) vs monotherapy (BRAF inhibition). The characteristics of these trials are presented in Table 1.

**Table 1:** Main characteristics of eligible RCTs

Author year	Study type	N	Age, years	Male, no%	OS (%)	ORR (%)	PFS months	Mortality n	Treatment regimen
Long et al 2015	Phase III	211	55.0 (22‐89)	111 (53)	74	69	11.0	99	Dabrafenib (150 mg, bid) and trametinib (2 mg, qd)
	RCT	212	56.5 (22‐86)	114 (54)	68	53	8.8	123	Dabrafenib (150 mg, bid)and placebo
Ascierto et al 2016	Phase III	247	56 (23‐88)	146 (59)	74.50	70	12.3	5/247	Cobimetinib (60 mg, qd)and vemurafenib (960 mg, bid)
	RCT	248	55 (25‐85)	140 (56)	63.80	50	7.2	3/246	vemurafenib (960 mg, bid) and placbo
Flaherty et al 2012	Phase I/II	54	58 (27‐79)	34 (63)	79	76	9.4	32	Dabrafenib (150 mg, bid) and trametinib (2 mg, qd)
	RCT	54	50 (18‐82)	29 (54)	70	54	5.8	49	Dabrafenib (150 mg, bid) only
Larkin et al 2014	Phase III	247	56 (23‐88)	146 (59)	81	68	9.9	34	Vemurafenib (960 mg, bid) and cobimetinib (60 mg, qd)
	RCT	248	55 (25‐85)	140 (56)	73	45	6.2	51	Vemurafenib (960 mg, bid) and placebo
Robert et al 2015	Phase III	352	55 (18‐91)	208 (59)	72	64	11.4	100	Dabrafenib (150 mg, bid) and trametinib (2 mg, qd)
	RCT	352	54 (18‐88)	180 (51)	65	51	7.3	122	Vemurafenib only (960 mg, bid)
Dummer et al 2018	Phase III	192	57 (20‐89)	115 (60)	76	64	14.8	/	Encorafenib (450 mg, qd) and binimetinib (45 mg, bid)
	RCT	194	54 (23‐88)	109 (56)	/	52	9.2	/	Encorafenib (300 mg, qd)
		191	56 (21‐82)	111 (58)	63	41	7.3	/	Vemurafenib (960 mg, bid)
Dummer et al 2017	Advanced	258	/	/	/	66	12.9	/	Encorafenib (300 mg, qd) and binimetinib (45 mg, bid)
	RCT	86	/	/	/	50	7.4	/	Encorafenib (300 mg,qd)

Abbreviations: bid, twice a day; N, number of enrolled patients; ORR, overall response rate; OS, overall survival; PFS, progression‐free survival; qd, once a day; RCTs, randomized controlled trials.

### 3.2 Overall survival and progression‐free survival and treatment response

**Figure 2:**
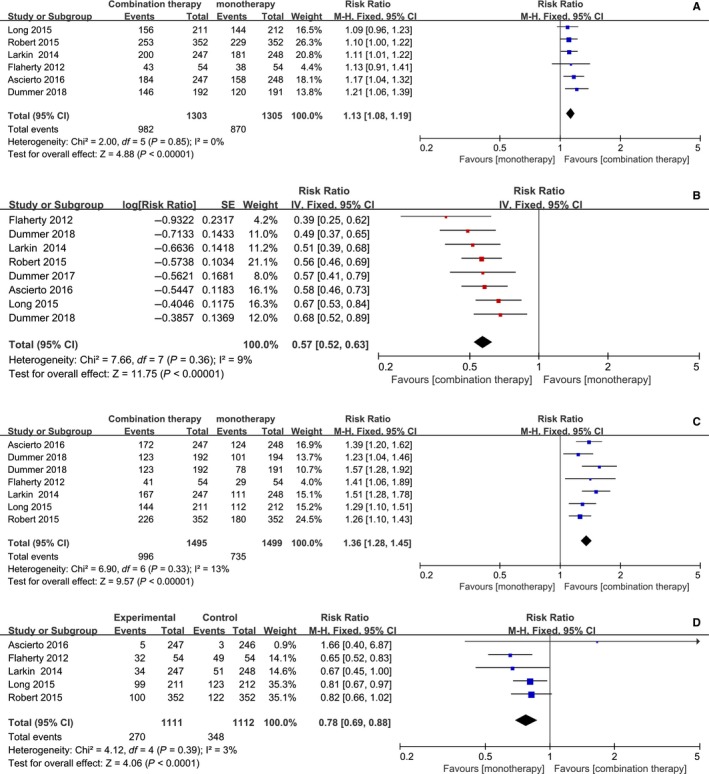
Forest plots of RR associated with combination therapy vs monotherapy. (A) OS (overall survival); (B) PFS (progression‐free survival); (C) ORR (overall response rate); (D) Mortality

The risk ratios (RR) for overall survival (OS), mortality, overall response rate (ORR), PFS were 1.13 (95% CI, 1.08, 1.19; *P* < 0.00001), 0.78 (95% CI, 0.69, 0.88; *P* < 0.0001), and 1.36 (95%CI, 1.28, 1.45, *P* < 0.00001), 0.57 (95% CI, 0.52, 0.63, *P* < 0.00001), respectively. Obviously, the *P* value of four outcomes indicated significantly statistical difference between combination therapy and monotherapy. Forest plots of OS, PFS, ORR, and mortality associated with combination therapy and monotherapy were showed in Figure 2.

### 3.3 Analysis of toxicity outcomes

**Figure 3:**
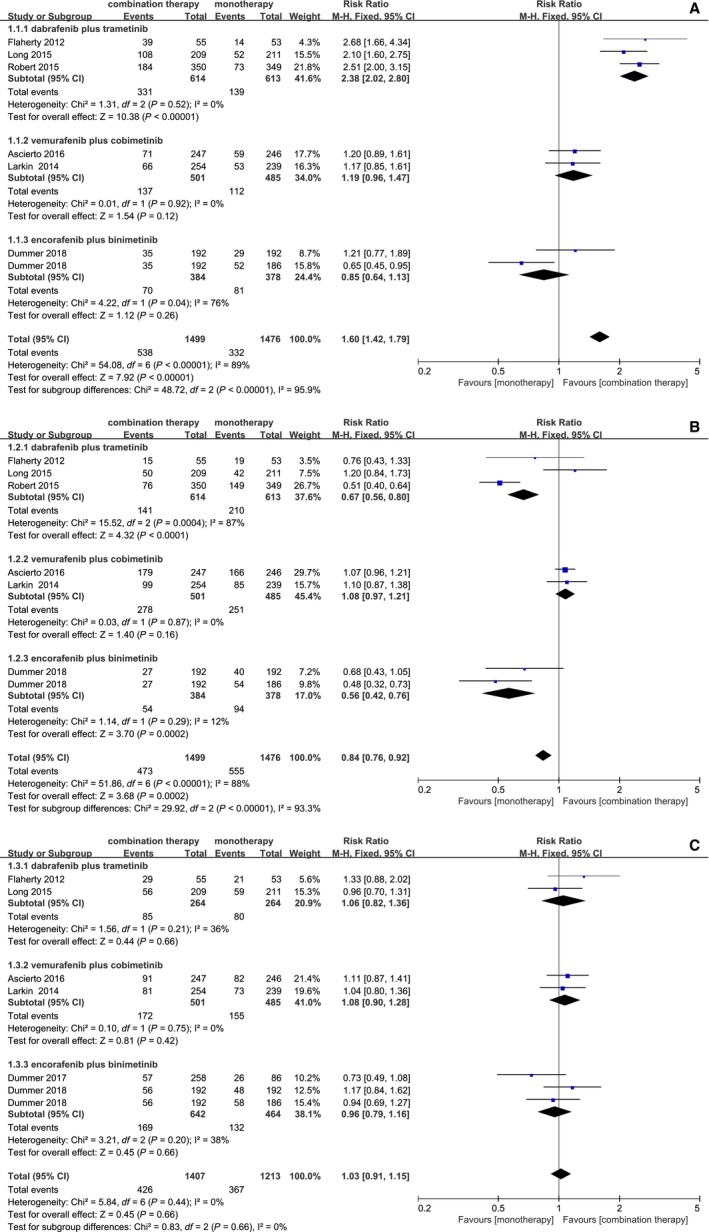
Forest plots of RR of adverse events for combination of BRAF and MEK inhibition vs BRAF inhibition. (A) Pyrexia; (B) Rash; (C) Fatigue

**Figure 4:**
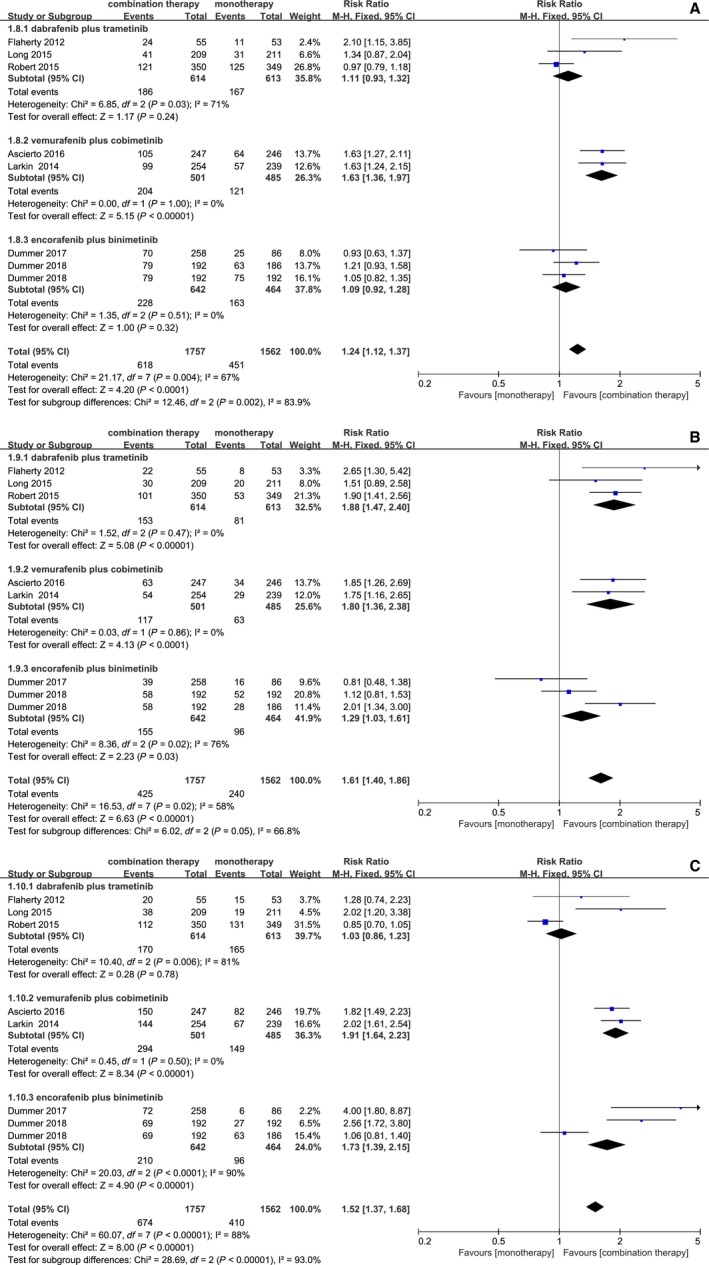
Forest plots of RR of adverse events for combination of BRAF and MEK inhibition vs BRAF inhibition. (A) Nausea; (B) Vomiting; (C) Diarrhea

**Figure 5:**
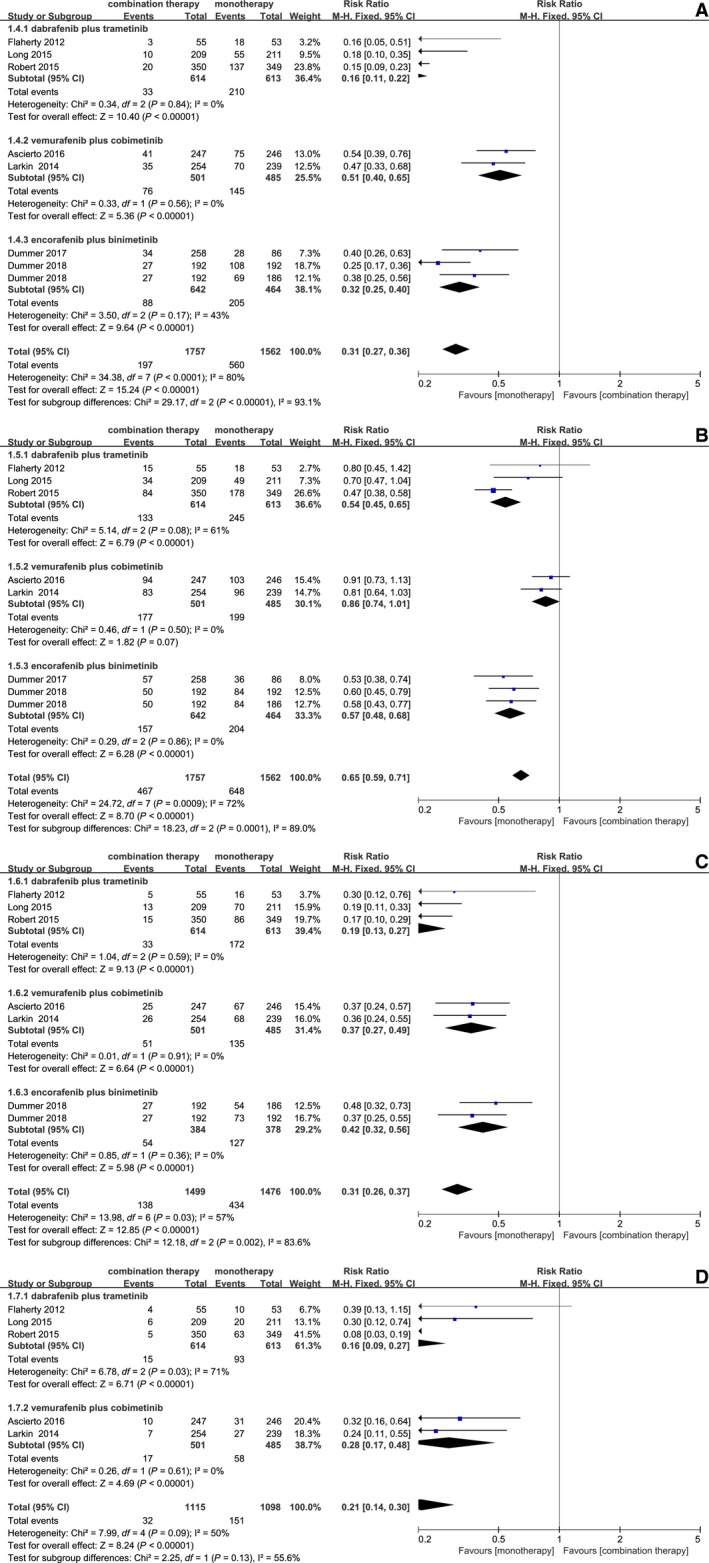
Forest plots of RR of adverse events for combination of BRAF and MEK inhibition vs BRAF inhibition. (A) Alopecia; (B) Arthralgia; (C) Hyperkeratosis; (D) CSCC

Combination therapy was related to a more frequent incidence of pyrexia (RR = 1.60; 95% CI, 1.42,1.79; *P* < 0.00001) and gastrointestinal events such as diarrhea (RR = 1.52; 95% CI, 1.37, 1.68; *P* < 0.00001), and vomiting (RR = 1.61; 95% CI, 1.40, 1.86; *P* < 0.0001), nausea (RR = 1.24; 95% CI, 1.12, 1.37; *P* < 0.0001), compared to monotherapy. Table 2 presented the incidences of some adverse events. However, with a dramatic toxicity event difference shown in data analysis, the result favored BRAF inhibition over combination treatment in dermatologic diseases, including alopecia (RR = 0.31; 95% CI, 0.27, 0.36; *P* < 0.00001), arthralgia (RR = 0.65; 95% CI, 0.59, 0.71; *P* < 0.00001), hyperkeratosis (RR = 0.31; 95% CI, 0.26, 0.37; *P* < 0.00001), and cutaneous squamous‐cell carcinoma (RR = 0.21; 95% CI, 0.14, 0.30; *P* < 0.00001). A similar incidence of rash and fatigue occurred in both combination therapy and monotherapy. However, there were significant heterogeneity in pyrexia (*I* ^2^ = 89%, *P* < 0.00001), diarrhea (*I* ^2^ = 88%, *P* < 0.00001), rash (*I* ^2^ = 88%, *P* < 0.00001), alopecia (*I* ^2^ = 80%, *P* < 0.0001), arthralgia (*I* ^2^ = 72%, *P* = 0.0009), nausea (*I* ^2^= 67%, *P* = 0.004), vomiting (*I* ^2^ = 58%, *P* = 0.02), and hyperkeratosis (*I* ^2^ = 57%, *P* = 0.03) (Figures 3, 4, 5).

### 3.4 Subgroup analysis

A subgroup analysis was conducted on account of significant heterogeneity in our analysis of adverse outcomes. According to combination drugs, all trials were classified into three subgroups: (1) combination of dabrafenib and trametinib vs dabrafenib or vemurafenib; (2) combination of vemurafenib and cobimetinib vs vemurafenib; (3) combination of encorafenib and binimetinib vs encorafenib or vemurafenib. Among subgroup analysis for adverse outcomes, the group dabrafenib and trametinib showed obvious heterogeneity in nausea (*I* ^2^ = 71%, *P* = 0.03), diarrhea (*I* ^2^ = 81%, *P* = 0.006), cutaneous squamous‐cell carcinoma (*I* ^2^ = 71%, *P* = 0.03), arthralgia (*I* ^2^ = 61%, *P* = 0.08). The group encorafenib and binimetinib had significant heterogeneity in diarrhea (*I* ^2^ = 90%, *P* < 0.0001), pyrexia (*I* ^2^ = 76%, *P* = 0.04), vomiting (*I* ^2^ = 76%, *P* = 0.02). In conclusion, the significant heterogeneity of adverse outcomes came from the groups dabrafenib and trametinib, encorafenib and binimetinib. The reason may be related to different control drugs.

### 3.5 Publication bias

**Figure 6:**
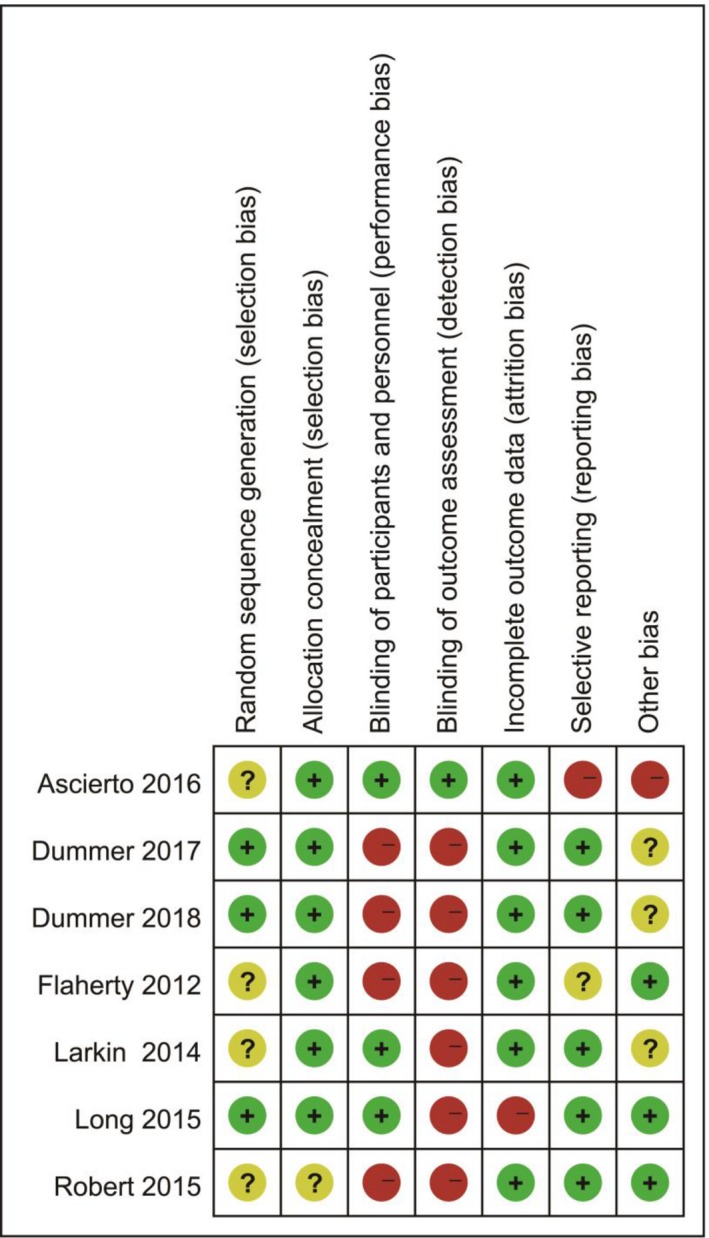
Risk of bias summary

**Figure 7:**
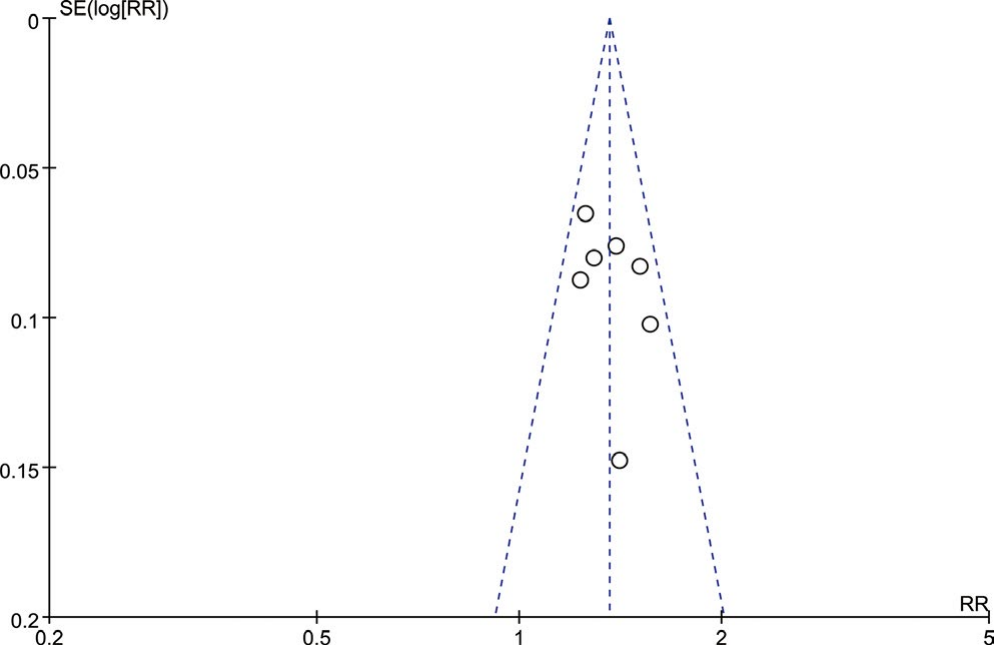
Funnel plot analysis for publication bias assessment

All the included studies were at low risk of bias, according to the Cochrane Collaboration tool. Four trials were blinded, whereas two trials were open‐label, but blinding was unclear in one study (Figure 6). Publication bias were also analyzed and showed a low risk. The funnel plot analysis is shown in Figure 7.

## 4 DISCUSSION

Melanoma is a highly mutated malignancy with poor prognosis.^20^ About half of all melanoma patients harbor an activating BRAF mutation.^21^ Our meta‐analysis suggests that BRAF inhibition in combination with MEK has a protective effect compared to BRAF inhibition alone owing to the better overall survival, response rate and reduction the risk of death events. And skin‐related adverse events such as hyperkeratosis, cutaneous squamous‐cell carcinoma were less compared with monotherapy. However, the occurrence rate of gastrointestinal events was higher in combination therapy. Pyrexia was also at a higher rate in doublet therapy of BRAF and MEK inhibition. The results were similar to former meta‐analysis.^17^

Combination therapy of MEK and BRAF inhibition provided a better overall survival and response rate. Theoretically, MEK and BRAF inhibitions restrain tumor cell proliferation in the way of inhibiting gene expression directly, which plays a necessary role in MAPK pathway.^22^ In addition, BRAF and MEK inhibition could affect CD8^+^ T cell of immune system, consequently promoting the expression of melanoma antigens and enhancing T‐cell cytotoxicity.^23^ However, resistance to BRAF inhibitions limited the onset time, which restricted the duration of effective treatment.^24^ Mechanisms of resistance were fasten on MAPK (mitogen‐activated protein kinase) pathway reactivation, gene expression change including acquired overexpression of upstream NRSA and MEK mutations, amplification or alternate splicing of mutant BRAF, reactivation and autophagy of ERK (extracellular regulated protein kinases),^25^ COT and MLKs overexpression.^26^ Besides, tumor microenvironment change such as microRNA and aryl hydrocarbon receptor (AhR) transcription factor has been found in BRAF resistance.^27^, ^28^ Immune microenvironment change such as increasing PD1+ melanoma cells was also related to tumor recurrence.^29^ These findings suggested that the occurrence of acquired resistance could be prevented or delayed in combination therapy, which included downstream target inhibition and BRAF inhibition. Consistent with our study, a number of studies demonstrated that combination therapy would be more efficient to inhibit acquired resistance in the process of development and showed better survival benefit.^21^, ^30^, ^31^ Studies of recent development and obstacles of melanoma with BRAF and MEK inhibition as MAPK pathway inhibitor significantly overcame acquired resistance and had a higher survival rate and a more durable response rate.^32^, ^33^ It had been reported that combination of MEK and BRAF inhibition had improved the quality of life of patient with metastatic melanoma.^34^

In our meta‐analysis, the occurrence of skin events was significantly reduced in the BRAF and MEK group. A certain percentage of patients acquired these events during therapeutic process.^35^ The possible explanation was recognized as reactivation of MAPK pathway, the way resulting in explosive cell growth.^36^, ^37^ At present, there was a study focusing on spectrum of cutaneous adverse events during the treatment of melanoma, although the study had only reported encorafenib and binimetinib, it actually demonstrated a lower rate in skin toxicity with combined MEK and BRAF inhibition.^38^ Meanwhile, the study showed that diverse cutaneous events emerged various degrees in monotherapy and combination group, suggesting a more complex mechanism in this kind of adverse event and a harder improvement to stop skin untoward events. Cutaneous adverse events of melanoma therapies were described in a review which was in common with our results.^39^

However, combination therapy brought out some additional toxicities. Gastrointestinal events, including nausea, diarrhea, vomiting were found in our meta‐analysis. Knispel et al had ever emphasized the significant role of these side effects related to life quality although the effects were moderate, reversible, and can be managed.^40^ Livingstone et al had also demonstrated the same result.^41^ Pyrexia was found at a higher risk in our meta‐analysis. A study concentrated on pyrexia confirmed that pyrexia occurred the first 3 weeks of treatment. Frequency and recurrence were the clinical features of pyrexia.^42^ Based on these studies and the results of our analysis, it was not an accident and it might be a focus we should pay attention to. The additional gastrointestinal events may be relevant to MEK inhibition as there were reports demonstrating that diarrhea was a common toxicity event.^22^, ^43^ There was no doubt that these additional toxicities would reduce life quality to some degree. However, more researches were needed to find out whether it was significant or not in guiding drug use.

In general, our meta‐analysis included the latest seven randomized controlled trials concerning metastatic melanoma patients. Furthermore, we analyzed effects and safety of combination therapy by comparing with single‐agent BRAF inhibition. However, limitations can be seen in our meta‐analysis. The first, dose standard and type of drug were not separated for more precise analysis, which may be the main origin of heterogeneity. The second, the reported adverse events varied from one to another articles, along with diverse evaluation criteria.

## 5 CONCLUSION

In conclusion, combination of BRAF and MEK inhibition achieved better survival benefit compared with single drug BRAF inhibition. Besides, skin‐related events were less but gastrointestinal events were more in combination therapy. In addition, more randomized controlled trials are required for further research.

## CONFLICT OF INTEREST

None declared.

6

**Table 2:** Toxicity events

Toxicity events	Number	Pyrexia	Fatigue	Rash	Nausea	Vomiting	Diarrhea	Alopecia	Arthralgia	Hyperkeratosis	CSCC
Long 2015	N_combo_ = 209	52%	27%	24%	20%	14%	18%	5%	16%	6%	3%
	N_mono_ = 211	25%	28%	20%	15%	9%	9%	26%	23%	33%	9%
Ascierto 2016	N_combo_ = 247	29%	37%	73%	43%	26%	61%	17%	38%	10%	4%
	N_mono_ = 246	24%	33%	68%	26%	14%	33%	31%	42%	27%	13%
Flaherty 2012	N_combo_ = 55	71%	53%	27%	44%	40%	36%	5%	27%	9%	7%
	N_mono_ = 53	26%	40%	36%	21%	15%	28%	34%	34%	30%	19%
Larkin 2014	N_combo_ = 254	26%	32%	39%	39%	21%	57%	14%	33%	10%	2.7%
	N_mono_ = 239	22%	31%	35%	24%	12%	28%	29%	40%	28%	11%
Robert 2015	N_combo_ = 350	53%	/	22%	35%	29%	32%	6%	24%	4%	1%
	N_mono_ = 349	21%	/	43%	36%	15%	38%	39%	51%	25%	18%
Dummer 2018	N_combo_ = 192	18%	29%	14%	41%	30%	36%	14%	26%	14%	/
	N_enco_ = 192	15%	25%	21%	39%	27%	14%	56%	44%	38%	/
	N_vem_ = 186	28%	31%	29%	34%	15%	34%	37%	45%	29%	/
Dummer 2017	N_combo_ = 258	/	22%	/	27%	15%	28%	13%	22%	/	/
	N_mono_ = 86	/	30%	/	29%	19%	7%	33%	42%	/	/

Abbreviations: combo, combination therapy; CSCC, Cutaneous squamous‐cell carcinoma; enco, encorafenib alone; mono, monotherapy; vem, vemurafenib alone.
